# Supplementary material for: Are carbon emissions trading and green financial instruments synergistic? -Comprehensive quantitative research based on content analysis
Source: PLoS One. 2024 Mar 7;19(3):e0298601. doi: 10.1371/journal.pone.0298601 (PMC10919601; doi:10.1371/journal.pone.0298601)
Supplement: S1 Data — (ZIP) [file pone.0298601.s001.zip › Data/地级市Z_F工作报告.zip!/║╙▒▒/└╚╖╗╩╨/└┤╫╘╣1⁄2╓┌║┼í╛┬φ┐╦╩2╛▌═°í┐_2007.docx]

各位代表：

现在，我代表廊坊市人民政府，向大会作工作报告，请予审议，并请各位政协委员及其他列席人员提出意见。

2006年工作回顾

2006年是“十一五”起始之年，是攻坚突破、锐意进取的一年。一年来，在中共廊坊市委的正确领导下，全市上下围绕建设“实力廊坊、效率廊坊、和谐廊坊”，认真落实科学发展观，扎实推进“招商引资突破年”，胜利完成了市四届人大四次会议确定的各项任务，实现了开好局、起好步的目标。

回顾过去的一年，全市经济社会发展实现了历史性突破，集中体现为三大亮点：一是以富士康等超大项目落户为标志，廊坊的项目承载力迈上新台阶，初步构筑了我市产业发展新格局；二是以实施农村“六项工程”为标志，社会保障体系向农村全面延伸，老百姓有了一个初步的“安全网”；三是以市区“十大工程”全面启动为标志，城市建设进入新阶段，中心城市在全市城镇群中的地位进一步凸现。

回顾过去的一年，全市经济社会发展质量提高、增速加快、后劲增强、协调并进，主要取得了六方面成果：

(一)经济增速与质量同步提高,综合实力进一步增强

主要指标增速跨入全省前列。地区生产总值、全部财政收入、地方一般预算收入(按省可比口径)、城镇固定资产投资、规模以上工业增加值、社会消费品零售总额、实际利用外资7项指标增速位居全省第二。全市地区生产总值完成722亿元，同比增长15.2%；全部财政收入74.4亿元，增长27.3%，其中地方一般预算收入完成30.5亿元，增长25.7%；城镇固定资产投资完成380.5亿元，增长47.5%；城镇居民人均可支配收入11263元，农民人均纯收入预计4950元，分别增长10.9%和7%。全部财政收入占地区生产总值比重首次突破10%，达到10.3%，同比提高0.9个百分点：税收占财政收入比重达82.8%，提高0.8个百分点；规模以上工业企业实现利税同比增长33.1%。

(二)项目建设跃上新台阶，产业结构显著优化

大项目建设取得重大突破。着眼落实科学发展观，以项目为抓手，加快产业结构调整。全年实施千万元以上项目1264项，同比增加437项，完成投资353.7亿元，同比增长60%，其中亿元以上项目287项，增加140项。全市列入省重点建设项目103项，占全省的18.3%。重点项目个数全省第一。以华为、富士康、京东方、中兴四大项目为主体的信息产业制造基地正在形成。华为、京东方列入全省十大产业支撑项目，富士康一期项目将于今年8月份投产，中科廊坊科技谷将建成区域创新服务平台和高科技产业化基地。

园区成为项目引进的主战场。实现每个县(市、区)都有省级园区。全市引进的千万元以上项目，70%以上落户园区。廊坊开发区有望升格为国家级开发区，龙河园区成为全省首家高新技术产业示范区，进入省级循环经济园区行列。争取国家开发银行贷款4.77亿元，用于园区基础建设，园区承载能力不断增强。

六大主导产业发展壮大。电子信息、汽车零部件、木材加工及家具制造、金属制品、食品加工产业实现增加值126.8亿元。会展旅游业全年举办大型展会48个，接待国内外游客467.2万人次，旅游收入36.3亿元。五星级酒店建设进展顺利，国际饭店B座及配套设施竣工，澳美基业、恒盛阳光等项目正在加紧建设。

(三)发展环境日益优化。软实力进一步提升

基础设施建设加强。全市共完成投资34.3亿元，其中，高头220千伏输变电工程、移动和网通扩容工程等32个项目竣工，密涿高速公路一期、燕郊和香河污水处理厂等16个项目开工建设。

市区“十大工程”进展顺利。25个项目竣工，9个项目在建，开竣工项目占总数的63.3%，实现了“一年一变样”，为“三年大变样”开了个好头。人民公园改建后免费向市民开放，lO个综合市场改造、金泰小区一期工程相继竣工，城市水系、市体育场等工程取得实质性进展。南外环二期、金光道西延等13条道路建设全部完工，创造了市区道路建设里程最长、投资最多两个“第一”。此外，完成银河路、文化艺术中心二期等园林绿化工程，市区绿化覆盖率达到45%。建设改造垃圾中转站18座，垃圾处理率达到95%。

软环境建设扎实推进。信用体系、人才高地、标准化、金融服务体系建设取得新成果。完成中小企业基础信用数据录入，信用成果在项目招投标等领域开始应用。全年引进博士40名、硕士440名、中高级人才661人，人才密度达到12.72%。通过IS09000认证的企事业单位新增38家，累计达到1047家。全市金融机构贷款余额比年初增加88.4亿元，同比多增31亿元，增量居全省第三；存贷比达65.2%，高出全省9.2个百分点。全年发放中小企业联保贷款9亿元，缓解了中小企业担保难问题。霸州成立小额放贷公司，当年放贷1.65亿元。

城市创建取得新成果。先后被命名为“全国绿化模范城市”、“国家园林城市”、“国家环保模范城市”和“国家节水型城市”，再次被评为“国家双拥模范城市”。

(四)新农村建设扎实推进，农民享受到更多实惠

“六项工程”取得重大进展。围绕解决农民最现实、最紧迫的生活问题，让农民病有所医、老有所养，吃干净水、走柏油路，子女有学上、吃穿有保障，实施农村“医、学、路、水、保、养”六项工程。医疗惠民工程，深入推进三河农村新型合作医疗试点，启动香河、霸州两个试点，农民参合总数达到84.5万人，9.8万人得到补偿。投资6032万元，新建、改造乡镇卫生院38个。义务教育“控辍保学”工程，发放“两免一补”专项资金1250万元，5.1万名中小学生从中受益。村村通油路工程，投资1.3亿元，新建通村油路773公里，新增通油路村298个，在全省第一批实现村村通油路。人畜饮水安全工程，投资7023万元，解决了256个村街、21.4万人的饮水问题，全市自来水入户率达到88%。社会保障工程，实现了农村低保应保尽保，共发放低保金1200万元。保障人数4.48万人。“五保户”集中供养工程，投资1.4亿元，新改扩建敬老院34所，集中供养率达到58%，在全省率先实现50%以上集中供养目标。

农业农村工作稳步推进。不折不扣地落实各项惠农政策，全面取消农业税。粮食生产再获丰收，总产量170万吨。奶牛存栏7.1万头，产奶量突破20万吨。完成造林任务30万亩，绿化村庄422个。农业开发项目投资1.1亿元，列全省第二。农业产业化经营率达到58%，省级重点龙头企业达到20家，永清、文安、大厂、三河列入首批省级农业科技工程示范县，大厂成为省级农产品加工基地县。实施“万村千乡市场工程”，建成标准化“农家店”700家。建成文明生态村400个。

(五)改革开放有新突破，经济发展更富活力

各项改革深入推进。行政审批制度改革，建立了涉民、涉企和重大项目三个“一条龙”服务体系，审批事项按时办结率99.9%。国企改革，初步理顺了市属经营性国有资产监管体制，完成恒宇工具、外贸包装等21家企业改制，退出国有资本9185万元，调整职工劳动关系2580人。社会和公用事业改制面达到30%。

民营经济快速发展。全市民营经济单位达到11.4万个，完成增加值579亿元，上缴税金32.9亿元，增长39.7%。新增“梅花”、“福成”两个全国驰名商标，“华日”家具被评为中国名牌产品。

招商引资成效明显。全年实际利用外资3.15亿美元，增长19.3%；引进内资123.3亿元，增长40%。“5·18”国际商务节和“9·26”农交会圆满成功，市场化、专业化、国际化程度进一步提高。外贸进出口总额16.3亿美元，增长45.5%。

(六)经济社会协调发展，和谐廊坊建设取得新成绩

民心工程扎实推进。全市新增就业岗位3.5万个，安置9157名下岗失业人员再就业。城镇登记失业率1%，低于控制指标2.5个百分点。农村劳动力向非农产业转移24万人。住房公积金新增缴存职工3.4万人，同比增加74.4%，增幅居全省第一。五大保险新增参保人员12万人，其中养老、医疗、生育、工伤保险扩面在全省名列前茅。食品药品放心工程深入推进。

社会事业健康发展。跨入河北省首批“创新型城市试点”行列，被科技部批准为“国家信息产业基地”。全市高新技术产业增加值达到54.6亿元，同比增长21.3%。投入4亿多元，改扩建26所高中和中等职业学校，高中阶段适龄人口毛入学率达到76.7%。三河、广阳、香河、霸州、文安全部消除农村小学D级危房。“文化信息资源共享工程”走在全国前列，“中国之声”特别节目“走进廊坊”在全国引起轰动，廊坊文艺成果出版工程成效明显，“和谐廊坊·市民文化大讲堂”反响良好，市博物馆建成开馆，市疾控中心投入使用。在省第十二届运动会上取得好成绩。信访、物价、统计、审计、民政、防震减灾、民族宗教、外事侨务、广播电视、新闻出版、档案、地方志、人防、气象等工作均取得新进展。

人口资源环境工作得到加强。人口自然增长率控制在5.5‰ 。启动了“国家生态市”创建工作。跻身全国7家“保护臭氧层示范省市”。完成污染物排放总量削减任务，IS014000环境管理体系得到有效保持和运行。市区空气质量二级以上天数332天。

民主法制建设取得新进展。坚持依法行政，全面落实《行政许可法》，自觉接受法律监督和民主监督，人大代表建议和政协委员提案办理答复满意率97.7%。加强廉政建设，深入开展行风评议活动。严格落实安全生产责任制，全市发生事故数量、死亡人数分别下降16.9%和14.8%。积极推进“平安廊坊”创建，深入开展严打整治和综合治理，社会治安保持稳定。

各位代表，过去的一年，是廊坊经济优质高速发展的一年，是社会和谐进步的一年。我们不仅完成了四届人大四次会议确定的各项任务，还在诸多方面实现突破，赢得了超出预想的成果。今天的廊坊大地，到处都涌动着立大志、创大业的激情，营造起团结进取、干事为民的良好氛围，形成了科学发展、和谐发展、竞相发展的大势。

各位代表，成绩来之不易。这些成绩是在省委、省政府和市委正确领导下取得的，是历届班子打基础、造环境成果的延续，是“全党抓经济，重点抓工业，集中精力上项目”的集中体现，是全市上下戮力同心、顽强拼搏的结果。在此，我代表廊坊市人民政府，向人大代表、政协委员、各民主党派、工商联和社会各界人士以及全市人民，向驻廊中省直单位、人民解放军、武警官兵和政法干警，向关心、支持廊坊的中外客商与朋友们，表示衷心的感谢!

看到成绩的同时，我们也清醒地意识到，廊坊经济社会发展中还存在着不少困难和问题：一是经济总量偏小、支柱产业不突出、产业结构不合理等状况并没有根本改变；二是社会事业欠账较多，群众关注的热点难点问题还没有完全解决好；三是随着一批超大型项目落户廊坊，城市功能、产业配套、行政服务等方面不适应的问题更为突出，这是一个新的亟待破解的问题。对此，我们一定高度重视，认真加以解决。

2007年工作目标与要求

2007年是我市经济社会发展的关键一年，是本届政府任期的最后一年。当前，我们面临着良好的形势，以往的发展和2006年的良好开局，为我们奠定了坚实基础；省委、省政府提出建设沿海经济社会发展强省，为我们指明了努力方向，也提供了难得机遇。天时、地利、人和俱备，如果我们奋力一搏，做好2007年的工作，就可能象东莞、昆山那样，开创廊坊发展的新天地；如果劲头不足、步伐不快，就会错过这一轮发展机遇，丧失来之不易的好局面。我们一定要倍加珍惜当前政通人和、蒸蒸日上的好形势，不辜负省委、省政府和全市人民的期望，围绕乘势而上、率先发展的主题，全市上下更加团结和谐、更加集中精力、更加奋力工作、更加扎实求效，挑起“三副担子”，为建设沿海经济社会发展强省多做贡献。

2007年工作的总体要求是：以邓小平理论和“三个代表”重要思想为指导，全面贯彻党的十六届六中全会、省七次党代会和市四次党代会精神，坚持以科学发展观为统揽，按照“突出好、追求快，好中求快”的发展理念，乘势而上、率先发展，全力推进项目质量提高年，更加注重经济增长质量，更加注重环境质量，更加注重人民生活质量，进一步加快工业化、城镇化、农业产业化进程，大力推进城乡协调发展，全力建设“实力廊坊、效率廊坊、和谐廊坊”。

2007年全市经济社会发展的主要预期目标是：地区生产总值增长14%以上；全部财政收入增长22%以上，其中，地方一般预算收入增长19%以上：全社会固定资产投资增长25%以上，其中，城镇固定资产投资增长30%以上；规模以上工业增加值增长20%以上；城镇居民人均可支配收入增长10%以上，农民人均纯收入增长7%以上；城镇新增就业3.4万人，城镇登记失业率控制在3.5%以内；单位生产总值能耗下降4.4%，主要污染物排放总量减少3%。

做好今年工作，必须把握以下几个方面：

——坚持“突出好、追求快”。目前廊坊已经走上科学发展、和谐发展之路，经济社会发展总体上是好的，我们必须保持这种“好”的发展模式，把区位优势变成竞争优势，确保可持续发展。同时，廊坊目前的主要矛盾仍然是总量小、实力弱，我们必须保持超常的增长速度，实现乘势而上、率先发展。没有“好”的发展模式，就可能断送廊坊美好的未来；没有“快”的增长速度，就不能有效化解各种现实矛盾和问题。因此，我们要坚持“突出好、追求快”，把“好”与“快”统一于廊坊崛起的全过程。

——注重项目质量。保证项目质量，就是保证经济质量和环境质量。项目质量不高，调优经济结构、转变增长方式、建设环境友好型社会，就会成为一句空话。要纠正个别地方在项目工作中急功近利、讲数量不重质晕的做法，坚决拒绝高消耗、高排放、低效益的项目。

——注重环境质量。环境是第一竞争点，抓环境就是抓竞争力。要不断完善城乡基础设施，优化基础环境，不断增强城市竞争力。

——注重民生质量。社会和谐，民生是基础。改善民生质量，缩小层次差距，让百姓得到更多实惠，是发展的根本目的，是社会和谐的重要保障。必颏一手抓发展，一手解民忧，让群众共享发展成果。

——推进城乡协调发展。以城带乡、城乡统筹，是经济社会发展到一定阶段的必然要求，也是新农村建设的重要途径。要统筹配置公共资源，使公共财政的阳光覆盖农村。把新农村建设与工业化、城市化结合起来，实现城乡协调、共同进步。

2007年工作措施

做好2007年的工作，关键是围绕建设“实力廊坊、效率廊坊、和谐廊坊”目标，突出壮大经济实力和解决社会现实问题“两条主线”，把握“三大抓手”，做好“十方面重点工作”。

把握“三大抓手”：

建设一批牵动全局的大项目。扎实开展“项目质量提高年”，重点抓好10个超10亿元项目、3个超100亿元项目，争列3个省重大产业支撑项目。力争重大产业支撑项目数、省重点项目数、争取增量土地指标保持全省第一。

落实一批关系民生的实事。突出解决“百姓急需、社会难点、历史欠账”三方面问题，办好低收入家庭住房困难、孤儿救助、食品药品安全等18件实事，把力量用到最困难的地方和最需要的人身上，把百姓的“安全网”织得更密、更牢，让群众的生活质量和环境更高、更好。

谋划实施一批事关长远的战略举措。以“两带一轴一中心”为主体，优化生产力布局；以万庄生态新城和城市次中心建设为重点，拓展提升城市平台；以“北三县经济发展一体化”、村庄布局调整和强镇扩权为导向，推进城乡协调发展；以加快中科廊坊科技谷和清华生物科技园建设为契机，推动科技创新。

做好“十方面重点工作”：

(一)开展“项目质量提高年”，讲求项目综合效益

“项目质量提高年”是今年经济工作的核心，是“大项目攻坚年”、“招商引资突破年”的深入推进，是今年政府工作的主要抓手。以提高项目质量为重点，促进项目质量、数量和规模的全面提升。全年力争实施千万元以上项目1500个，其中，投资亿元以上项目300个，同比分别增加300个和40个。

大幅提高项目服务水平，确保大项目顺利实施。按照世界500强企业的需求和标准，改进和提高项目服务。认真总结和推广富士康项目服务模式，对重大项目实行一个窗口对外，一个专门班子协调，全天候、零缺陷服务，全过程跟踪，全方位对接，保证重大项目无障碍实施。确保华为、富士康、京东方三大项目年内投产，科技谷、中兴、成功大广场等项目开工建设。

提高项目“门槛”，谋划引进一批高质量项目。严把项目准入关，按照“一大两低三高”(投资规模大，低消耗、低污染，高科技含量、高效益、高关联度)标准，面向世界500强、跨国公司、国家级大企业和民营百强企业，全力攻坚，引进一批创新能力强、拥有自主品牌的高质量项目。

围绕产业聚集抓项目。重点建设信息产业项目群。围绕落户的信息产业龙头项目，实施专门招商，谋划引进一批关联配套项目。通过项目联动，上下游配套，把大项目发展成大基地。完善信息产业发展规划，整合光伏产业和LED(半导体发光二极管)“双绿”产业链条，引进一批重量级项目，打造廊坊信息产业品牌。2007年，电子信息产业规模和集中度明显提升，销售收入新增70亿元，成为打造河北第四大战略支撑产业的生力军。

着眼提高产业竞争力，抓住新兴产业上项目。在继续壮大六大主导产业规模的同时，围绕生物医药、新材料、物流、服务外包、文化创意等新兴领域，推行我市信息产业发展模式，从零开始，从规划人手，以园区为依托，打造新的产业竞争点。生物医药、新材料产业，力争引进1—2家龙头项目，壮大产业集群；物流产业，重点抓好法国佛玛、国际区域性物流产业园等项目建设；服务外包产业，制定鼓励措施，加强产业招商，争取引进3—5家跨国公司服务外包企业，积极申列国家级服务外包基地；文化创意产业，充分利用域内及京津人才、文化资源优势，对重点聚集区域和产业领域进行研究和规划，争取快速启动、重点突破。

(二)突破三大瓶颈，打造发展新优势

重点围绕体制机制、土地、资金三个方面。逐一破解难题，开辟发展空间。

推进区域经济一体化。组建市城乡规划局，对全市辖区统一规划。按照未来廊坊城市群的设想，启动“大四点”前期工作，统一谋划基础设施建设。做好“北三县经济发展一体化”规划，建立协调推进的组织和机制，研究“北三县”战略定位、空间布局和产业分工。创新财政体制，实现投资分担、属地征收、利益共享。

积极稳妥地推进强镇扩权。筛选3—5个经济实力强、发展潜力大的建制镇，作为全市扩权镇试点，扩大权限，创新机制，激发活力，设立相对独立的一级国库，提高镇政府的决策自主性，促进各项事业发展，发挥其辐射带动作用。

启动村庄整理试点。实施“三个一批”：一是转变一批，城中村和位于城郊的村庄，与城镇一体规划、一体建设，加快农村向城市、村组向社区、农民向市民转变；二是提升一批，具有较强产业基础和带动能力的村庄，联手周边村庄，集中规划、联片建设，向小城镇发展；三是改善一批，具有一定人口规模的村庄，立足现有基础，健全公共设施，改善人居环境，逐步发展为中心村。不断提高城乡一体化水平，逐步实现城乡土地统一规划，路网、自来水管网、污水管网等基础设施向农村延伸，劳动就业服务、社会保障、教育医疗、文化体育等各项事业全面对接。

科学集约用地。依据国家土地政策，创新工作思路，最大限度地解决项目空间制约。一是按照节约和保护土地资源的要求，力争上半年完成土地利用总体规划修编大纲评审，完成廊坊市区和各县(市)城镇规划修编。二是多争取和用好增量指标。积极争列省重大产业支撑项目和省重点项目，争取土地增量指标，对省下达指标统一调配使用，现有剩余规划指标向城镇、园区集中。三是深化存量挖潜。抓好旧城改造，加强闲置、低效利用土地治理及集体建设用地整理，拓展项目用地空间。四是节约集约用地。提高用地门槛，对投资不到位的不予供地。加强批后跟踪监管，对违约项目依法进行处罚。

打造资金洼地。充分发挥政府作用，建设一流金融生态环境，实现金融与地方经济互动双赢。一是增强金融对经济的支撑力。奖励对地方发展贡献突出的金融机构，提高信用担保能力，规范发展民间信贷，扩大小额信贷试点，鼓励农村金融创新；办好经济金融合作恳谈会，推进京津廊信贷一体化，加强与国家开发银行等金融机构的合作，争取更多域外资金。二是大力改善金融生态环境。加大行政推动，解决逃废债问题。全力支持农村信用社深化改革，确保早日兑付16.5亿元专项票据。三是努力建设金融服务基地。引进域外金融机构来廊设点开展业务，争取相关服务中心、基地建到廊坊。

(三)提升园区水平，增强项目承载力

围绕“规划、机制、人才”三要素，发挥廊坊、燕郊、霸州开发区带动作用，推进园区统一规划、合理分工，发挥各自优势，突出产业特色，建立项目转移利益分享机制，着力提升园区整体档次，实现“二次创业”。

调整园区战略布局，构筑园区发展新体系。按照国家土地政策，根据园区基础和发展规划，重点建设一个国家级园区、九个省级园区和一批乡镇产业功能区，形成布局合理、科学分工、互为依托的园区体系。强化廊坊开发区的龙头作用，争取早曰成为国家级开发区，实现廊坊出口加工区封关验收并引进龙头企业，整体实施“腾笼换鸟”和产业升级，重点发展总部基地、创新研发基地、服务外包基地和高档服务业基地。九个省级园区在规划提升上下功夫，重点发展高新技术产业和先进制造业，同时为廊坊开发区龙头企业提供配套服务。精心谋划产业功能区建设，争取每个重点镇都建成一个产业功能区，重点围绕六大主导产业，引进现代制造业和服务业项目，为省级园区提供支撑。

(四)改善基础环境，强化重大项目及可持续发展的支撑力

围绕降低企业运营成本，为投资者提供良好的生态环境和信用环境，以及有力的人力资源支撑，加大工作力度，增强廊坊可持续发展能力。

改善生态环境。市区新植乔木300万株，通过绿化改造、补植增绿消除市区裸露地面，加快启动廊坊植物园建设。实施廊大线、廊九线、廊霸线绿廊建设，两侧各建设100米绿化带，全部清除沿线违章建筑和违规项目，以绿色精品线连接城乡、贯通南北。

加强交通建设。一是完善路网规划。建立与京津路网对接合作机制，做好高速公路、干线公路、高速连接线、京津道路接口，以及“小三点”、“大四点”快速交通体系的整体谋划。二是启动6个高速及干线公路项目。其中，廊沧高速、唐通线香河至北京段、京沪高速连接线大城段力争上半年开工，密涿支线、廊涿高速永清及固安连接线力争年内开工。三是争取光明西道西延、西外环南延和郊区快速路北段等结转项目上半年竣工。

改善信用环境。加快信用体系建设，出台企业和个人信用管理等配套政策，引入权威机构进行信用等级评定，利用网络平台整合信用资源，在重点项目、市政建设、政府采购、追缴债务等方面广泛应用。力争2008年初步建成个人信用体系。

加速培养和整合人力资源。继续加大公务员、企业家、科技人员等人才引进和培养力度。探索建立人才与项目对接机制，搭建服务平台，围绕主导产业和重大项目开展专项人力资源服务。集中力量发展职业教育，支持企业自办、校企合办职教培训机构，建设一批校外实训基地，培养企业所需的技术工人，保障企业人力资源需求。

(五)推进市区“十大工程”，提升城市竞争力

区域经济竞争，首先表现为中心城市间的竞争。以推进市区“十大工程”为主体，不断提升城市功能，完善城市管理，打造城市品牌，强化对重大项目的支撑力。2007年，“十大工程”新开工22项，年底累计竣工45项，竣工总数占“十大工程”项目的83%。

提升中心城市整体功能。推动龙河、廊坊开发区两个城市次中心的规划设计，对商住、道路、文化、教育等功能配套项目进行高标准设计，力争下半年开始起步区建设。启动万庄生态新城开发，完成一期控制性详规并开工。加快环城水系建设，汛期之前，八干渠、大皮营引渠实现通清水目标。提升绿化美化水平，投资4400万元，重点实施城区出口迎宾线绿化提升、环路绿化改造、瑞丰公园、城市游园等7大绿化美化工程。打通建设路等7条半截路。廊坊大剧院开工建设。完成北昌、银河北路等6个综合市场建设。加快城中村改造，探索新的开发模式，采取政府主导、市场化运作办法，根据城市发展规划，打破城中村行政界限，进行区域性开发改造。争取用3年时间完成城中村改造任务。

提升城市管理水平。一季度全面启动城市网格化管理。实施“大拆违”、“大整治”，全面清理拆除规划区内违规违法建筑，整治店外经营、马路市场、洗车站点。统一规划，合理设置户外广告牌匾，拆除楼顶、墙体、柱式广告设施。成立城市管理治安执法支队，建立公安参与、城管执法的长效机制。

深入开展城市创建。围绕全国文明城市、国家卫生城市、全国科技进步示范城市创建，扎实工作，逐步建成。申报中国人居环境奖。申办2010年全国绿化博览会。大力推进生态市创建，三河、霸州争创省级环保模范城，其它县(区)建设生态县，乡镇一级建设环京津环境优美城镇群。

(六)开展新农村建设，促进城乡协调发展

着眼于城乡协调发展，以壮大农村经济和增加农民收入为核心，以促进农民创业、转移就业为主要抓手，大力发展现代农业，协调推进农村工业化、城镇化和农业产业化步伐。

加快农业产业化进程。2007年，全市农业产业化经营率达到59%。着力发展现代农业。调整农业生产结构，重点发展生态农业、设施农业，加强优质粮、棉等基地建设。落实“111”重点项目建设计划。市、县两级分别抓好10个产业化重点项目，力争1—2个项目列入全省10大重点项目。做大做强龙头企业。在省级龙头企业中优选1家申报国家级龙头企业。销售收入500万元以上农产品加工企业达到155家。壮大种养基地规模。重点实施“百万吨奶”工程，2007年奶牛总量达到10万头以上，产奶量30万吨。加强农民培训，培训农民30万人，转移农村劳动力10万人，劳务输出6万人。

大力开展全民创业。营造创业氛围，建立创业机制，培育创业文化，让创业者在舆论上有支持、社会上有声誉、法律上有保障。一是富村带动。选择创业基础好的村，组建富带穷结对村。从企业用工、创业指导、企业资金等方面人手，通过整合土地、人力等资源，实现互补双赢。二是企业带动。筛选重点企业，通过直接帮扶、项目外包、供应原料等方式，支持带动家庭创业。三是领导干部包村。市级抓十个，县级抓一百个，乡镇抓一千个试点村。四是打造创业平台。大力推进信用担保等多渠道融资，稳妥发展中小企业合作基金联合会，强化创业培训，全年新建3—4个初具规模的创业辅导基地。

扎实推进农村综合改革。积极稳妥地推进乡镇机构改革、农村义务教育改革和县乡财政管理体制改革。以清理乡村债务和减轻农民负担为重点，妥善解决农村税费改革遗留问题，确保农民负担不反弹。深入推进“万村千乡市场工程”。

加强文明生态村建设。新规划重点创建村300个，巩固一、二批900个村的创建成果，建成100个村民中心示范村，开展生态文化、整洁环境、卫生厕所、清洁能源、绿色用品进农家的“五进”活动。

(七)依靠改革创新，激发经济发展活力

着力破除经济社会发展的体制机制障碍，进一步提高效率、激发活力、壮大实力。

创新招商机制。加大招商引资考核力度，发挥招商基金的激励作用。以园区为招商引资主战场，以企业为招商引资主体，建立企业化专职招商机构，委托国内外专业机构和人员进行市场化招商，加快形成招商新体制。

强化重大活动的载体作用。进一步办好“5·18”国际商务节，不断提升其档次和影响力。继续创新“9·26”农交会，建设农业示范观光园，全方位展示廊坊高新农业发展成果，把“9·26”办成永不落幕的农交会。

推进国企改革和上市融资。深化国企改革，继续按照“双退出、三接续”的改制标准，推进亏困企业和“空壳”企业通过改制重组、破产淘汰等途径加快退出。加快优质企业上市步伐，充实上市后备资源，引导基础较好企业进行股份公司改造。

加大公共事业改革力度。研究制定鼓励社会办事业的政策，引导社会资金进入医疗、卫生、教育领域。加快推进城市道路、供水、排水、供热、污水处理等方面的改革进程。对国有资源统筹运作，探索资源转化为资产、资产转化为资本、资本转化为资金投入的有效途径，完善地方投融资体系。

(八)解决群众关心的三方面问题，织密城乡“安全网”

本着民生优先的原则，在深人推进农村“六项工程”的基础上，由点到面，重点解决“百姓急需、社会难点、历史欠帐”三方面问题，全力抓好18件实事，让发展成果惠及广大群众。

切实解决好“百姓急需”问题。

1.残疾人生活困难问题。一是提高贫困残疾人救助标准，实现应保尽保。二是各级财政投入2000万元资金，并积极动员各方面力量，全面解决920户贫困残疾人的危房、无房问题。三是加大残疾人职业技能培训力度，拓宽就业领域，解决好残疾人就业问题。四是市县两级投入资金450万元，建立特困残疾人突发急重困难救助基金。

2.孤儿救助问题。推广大城县孤儿救助模式，对全市682名孤儿实行集中供养、集中就学，享受城镇低保，从根本上解决孤儿的生活、教育等问题。

3.“五保户”集中供养问题。在继续抓好敬老院硬件建设的同时，规范敬老院各项服务标准，开展“星级敬老院创建活动”，提高管理服务水平。“五保户”集中供养率达到65%。

4.农村人畜饮水问题。解决160个村街、12万人的饮水问题。推广联村集中供水模式，抓好固安农村集中供水试点，建设大厂、香河、广阳、安次、文安、大城、固安7座农村水厂。用两年时间解决118个高氟村街的饮水安全问题。

5.低收入家庭住房困难问题。做好最低收入家庭住房保障工作，积极落实国家房地产开发“双限双竞”政策，完善经济适用房货币化补贴办法，加大对中低收入家庭住房货币补贴力度，扩大覆盖面。人均15平方米以下低保户住房补贴实现应保尽保。进一步加大住房公积金归集和贷款发放力度，扩大覆盖和受益保障面，为中低收入家庭提供政策性购房保障资金。

6.城乡居民和大中专毕业生就业问题。统筹城乡一体化就业，以城镇“零就业家庭”、失地农民为重点，开展“一户一就业”活动，今年解决城镇“零就业家庭”205户、425人。健全毕业生市场就业体制，完善就业服务体系，广开就业渠道，积极推行毕业生就业见习制度，建立3所就业见习基地，引导毕业生面向基层、面向企业、面向生产第一线就业创业，争取就业率保持在85%左右。

切实解决好“社会难点”问题。

7.居民生命财产安全保障问题。深入开展“打黑除恶”，打击盗窃机动车和“两抢一盗”等专项斗争，“两抢一盗”案件下降10%。完善社会治安防控体系，建立健全群防网络，加强专业巡防力量，严格流动人口和出租房屋管理。

8.农村电力、电信设施保护问题。各级公安机关加强对外来可疑人员和可疑物品的检查，配合工商部门，对废旧物品收购站点进行彻底清理，取缔无照经营，狠抓源头控制，采取治本之策，使盗窃破坏电力、电信设施案件下降30%。

9.食品药品安全问题。实施食品放心工程，加大专项整治力度。推行食品市场准入制度，在全市创建1100个让百姓放心的规范经营示范店、10个规范经营市场。全面提高食品药品监管水平，深入开展“食品安全监管年”、“药品市场整顿年”活动，加强监管网络和基础设施建设，逐步建立长效管理机制。

10.农民看病难、看病贵问题。在省批准9个试点的基础上，在广阳区和廊坊开发区推行农村新型合作医疗，实现农村新型合作医疗制度全覆盖。投资1000万元，改造22所乡镇卫生院，使全市乡镇卫生院医疗用房、装备水平基本达标。

11.城区旧小区改造问题。确定3—5个项目，加大对环境的综合整治，进一步完善基础设施，彻底解决旧小区人口密度大、居住环境差、与城市发展不协调等问题。

12.市区交通拥堵问题。加强对市区主要拥堵路段的治理。优先发展公共交通，增加站点和线路，延长运营时间。加快道路建设，缓解交通压力。

切实解决好“历史欠账”问题。

13.基础教育欠账问题。加大城乡基础教育投入，改善办学条件。消除全市农村中小学现有D级危房。加快扩建廊坊市第十四小学和第一实验小学，新建廊坊市第十五小学，完成管道局中学的改扩建工程，力争在市区启动一所标准化初中建设。采取有效措施，逐步解决“普九”欠账问题。

14.农村公路建设问题。巩固村村通油路成果，2007年改造农村公路400公里，用3—5年完成破损严重农村公路的改造。结合文明生态村建设，加大投入力度，实施村内道路改造提升工程。积极探索农村公路养护管理机制，确保农村公路的可持续发展。

15.农村水利基础设施薄弱问题。积极争取上级资金支持，调动县乡村的积极性，多方筹集资金，逐步加强农村水利基础设施建设。年内财政投资1200万元，维修和改造37座桥、涵、闸。

16.社区建设滞后问题。投入2162万元，力争54个社区工作用房达到300平方米标准，占社区总数的69%。建立“工作进社区”准入制度，实现社区管理规范化、制度化。加强城市社区卫生服务工作，建设社区卫生服务中心8家，社区卫生服务站16家。

17.公教人员收入差距问题。各县(市、区)在加快经济发展、壮大综合财力的同时，调整支出结构，把落实各项工资制度改革和津贴补贴政策放在第一位，按省要求落实规范津贴补贴标准。

18.农民文化生活匮乏问题。做好全国“文化信息资源共享工程”试点工作，建好管好农村文化信息设施。建设80个农村图书室和60个农村文化大院，不断丰富农民的文化生活。

(九)发展各项社会事业，加强和谐廊坊建设

深入推进科教兴市战略。重点抓好全国科技进步示范市和国家可持续发展示范区的创建工作，加快环北京高科技产业带建设，建设创新型城市；加大科技普及和推广力度。全市高新技术增加值增长50%以上。深人实施“控辍保学”工程，认真落实“两免一补”，为义务教育阶段所有农村学生，以及城市贫困家庭学生免除杂费。确保2007年基本普及高中阶段教育，适龄人口毛入学率达到85%。

积极发展文化、体育、卫生事业。力争市图书馆新馆“五.一”开馆。制定出台印刷包装业发展规划，逐步把我市建成“北方印包产业基地”。办好市五运会，完成省十二运会参赛项目，确保实现“金牌第五、总分第六”目标。加快市体育场建设步伐，一期工程年底竣工。加强政府公共卫生职能，大力发展城乡基层卫生服务，积极稳妥地推进公立医疗机构改革，鼓励和支持民营医疗事业发展。抓好市急救指挥中心和24个急救站建设，形成覆盖全市的医疗急救网络；抓好第三医院建设，上半年投入使用。完善医疗设施建设规划，启动投资亿元的市妇女儿童医院，开工建设投资27亿元的河北燕达医院和老年医疗养护中心，谋划建设投资23亿元的诚志生命科技肿瘤医院，以及投资10亿元的廊坊开发区综合性三级医院。

切实加强安全稳定。各级各部门及广大公务员想问题、办事情，都要设身处地为百姓着想，真心实意维护群众利益，在决策和工作的全过程都要增强安全稳定意识，从根本上消除各种安全稳定隐患。把安全稳定作为各级政府压倒一切的基础工作，认真履行“一岗双责”。安全生产突出“四强化一确保”，即强化监管网络、专项整治、企业责任、应急救援，确保继续降低安全事故、人员伤亡和事故损失，各项安全指标在全省最低。建设人民调解、行政调解和司法调解“三位一体”的大调解工作体系，年内各乡镇、街道都要建成一个调解中心，建立一支过硬的调解员队伍，健全一整套规范的调解机制，确保90%以上的矛盾纠纷解决在县以下，全年不发生影响稳定的群体性事件。进一步加强信访工作，深入实施“十百千”工程，着力抓基层、打基础，围绕污染、拆迁、征地，以及农民工利益等热点难点问题，加大工作力度，把矛盾和纠纷解决在萌芽状态。

大力实施可持续发展战略。积极推进河北省循环经济试点市建设，以及左各庄胶合板、大城保温建材等循环经济示范园区建设，重点培育10家清洁生产示范企业；开展创建节约型机关、企业、社区活动，推动社会循环式消费。加强节能降耗和污染治理，严格落实环保法律法规，开展环保专项行动，加大对环境违法行为的处罚力度，加快重点区域、流域和企业的治理，完成节能降耗和污染减排的约束性目标。加快廊坊开发区和县(市)污水处理场、垃圾处理场建设。不断提高人口计生工作水平，人口出生率控制在12%以内。控制人口机械增长。优化人口结构。

加强精神文明建设。塑造积极向上的城市精神，激发广大市民热爱廊坊、建设廊坊的热情，使城市始终充满活力、富有朝气。大力宣传公民基本道德规范，形成良好的社会风尚。深入开展法制教育、科普教育，强化国防教育，着力加强“双拥”和国防后备力量建设。

(十)创新工作机制，提高政府行政水平

全面完成今年目标任务，为建设河北第四大战略支撑产业做贡献，兑现本届政府的承诺，各级政府责任重大，使命光荣。面对新挑战和新任务，必须切实加强政府自身建设，不断提高行政能力，努力打造公信力高、执行力强、工作作风硬、群众信得过的政府。

推进项目服务能力提升。把超大项目落户作为全面优化发展环境的重要契机，向深圳看齐，向适应世界500强的标准冲击，把行政功能、办事效率、服务水准全面提上新水平。把对大项目的服务水平作为考核评价干部的重要标准，把部门职能和权力全方位运用到服务大项目上来，大力提高各级干部的服务能力和专业素质，形成亲商、近商、安商、富商的好局面。

推进政府管理创新。进一步清理行政审批事项和收费项目，现有行政审批项目和收费项目再削减20%以上。进一步拓展行政审批中心服务职能，把行政审批中心建成方便市民和企业的服务中心，为公众提供便民、利民的公共服务平台。全面推进行政权力公开透明运行，开通“廊坊市行政权力公开透明运行网站”，争取年内乡镇以上机关全部实行公开透明运行，将行政职权、工作流程、时限结果全部公开，让行政权力在阳光下运行。完善突发公共事件总体和专项应急预案，形成高效有序的工作机制。不断提高保障公共安全和处置突发事件的能力。

推进各项工作落实。为政之要贵在落实，政府的责任就是抓落实。本事高低看落实，业绩好坏看落实，干成事、干成大事是各级政府不懈地追求。为此，我们要进一步改进工作作风，把时间和精力放到抓落实上，放到攻坚克难、解决实际问题上。健全落实制度，强化落实机制，把重要事项分解到部门，落实到人头，做到事事有人管，件件有着落。明确工作标准和完成时限，张榜公布，登报明示，定期向社会通报。

推进工作提质提速。不断完善政府决策、落实、督导工作机制，建立机关效能量化、考核评估和责任追究机制，加大对市政府年度工作计划和重要工作部署的政务督查，发挥行政效能监察部门作用。继续开展行风评议，着力解决办事难、环节多、效率低的问题，对不作为、乱作为的人和事坚决查处、决不姑息。积极推行电子政务，加大电子政务数据资源整合和共享力度，完善政务信息网络平台，在各级政府机关形成政令通、节奏快、效率高的局面。

推进机关政风建设。我们的权力是人民给的。政府的一切工作，必须以人民群众的根本利益为出发点，用真情实感贴近群众，用真心实意服务群众，用真抓实干造福群众。坚持以德谋政、以廉立身，严格执行党风廉政责任制，保证权力干净运行。切实加强公务员队伍建设，大力实施公务员培训工程，不断提升全市公务员依法行政、服务经济和开拓创新的能力，坚持用发展统一思想、鼓舞士气，在推进事业中增进团结、凝聚人心，把政通人和、协调一致的工作氛围造得更浓，把士气高昂、激情奉献的工作干劲鼓得更足，用扎实的工作、突出的成果，确保本届政府各项任务圆满完成。

各位代表!我们已经站在廊坊发展新的历史起点，形势催人奋进，事业任重道远。让我们在中共廊坊市委的坚强领导下，团结和带领全市人民，万众一心，奋发图强，共同创造更加幸福的生活，努力开辟更加美好的未来，以优异成绩迎接党的十七大的胜利召开!
